# Targeted spatial proteomic analysis of CD8^+^ T- and myeloid cells in tonsillar cancer

**DOI:** 10.3389/fonc.2023.1253418

**Published:** 2023-11-17

**Authors:** Can Altunbulakli, David G. Jimenez, David Askmyr, Aastha Sobti, Sabine Swoboda, Lennart Greiff, Malin Lindstedt

**Affiliations:** ^1^ Department of Immunotechnology, Lund University, Lund, Sweden; ^2^ Department of Otorhinolaryngology (ORL), Head & Neck Surgery, Skåne University Hospital, Lund, Sweden; ^3^ Department of Clinical Sciences, Lund University, Lund, Sweden

**Keywords:** spatial proteomics, head and neck (H&N) cancer, tonsillar cancer, immune check point, cytotoxic T lymphocytes (CTL), myeloid cell

## Abstract

**Background:**

Tonsillar cancer is caused by high-risk human papillomavirus (HPV), tobacco smoking, and alcohol abuse. Aspects of the patient’s immune response to this disease have arisen as prognostic factors and treatment targets, reflecting differences in the type and protein expression profile of immune cells. Because tonsillar cancers are heterogenous lesions such data need to be spatially resolved.

**Methods:**

In this study, we aim to explore inter-patient and intra-tumoral sources of variation in tonsillar cancer using immunofluorescence and targeted spatial proteomics to interrogate a cohort of 105 patients. Furthermore, we assess prognostic factors and elucidate molecular targets. We have used CD8, CD11c, and Pan-cytokeratin (PanCK) to quantify and locate immune cells driving antigen-specific cellular immunity. Guided by immunofluorescence information, we selected 355 CD8^+^, CD11c^+^, or PanCK^+^ areas inside and outside (i.e., stroma) cancer-cell islets, to quantify 43 immune-related proteins using digital spatial profiling.

**Results:**

Quantitative analysis of immunofluorescence in combination with clinical data revealed that the abundance of total CD8^+^ cells and CD8^+^ cells infiltrating cancer-cell islets, respectively, were associated with higher 5-year disease-free survival and overall survival, independently of HPV-status and clinical stage. Comparison of CD8^+^ cells inside and outside cancer-cell islets revealed an upregulation of effector CD8^+^ T-cell and immune checkpoint molecules in the former. Among these, the expression of PD-L1 by CD8^+^ T-cells was associated with lower all-cause mortality in a univariate proportional hazards model. Similarly, a comparison of tumor boundary and stroma CD11c^+^ cells showed upregulation of both co-stimulatory and immune checkpoint molecules with proximity to tumor cell islets.

**Conclusion:**

Our findings highlight the relevance of analyzing aspects of tumor micro-architecture in the search of prognostic markers and molecular targets for tonsillar cancer. The abundance of intra-tumoral CD8^+^ T-cells can be considered a positive predictive marker for tonsillar cancer, while the significance of PD-L1 expression by intra-tumoral CD8^+^ T-cells warrants further evaluation. Location-based differences in CD8^+^ and CD11c^+^ cells suggest an immune cell-altering effect on the tumor microenvironment, and grant new insight into which cells that can be targeted by novel therapeutic agents.

## Introduction

Tonsillar cancer, i.e., squamous cell cancer localized to the palatine tonsils of the oropharynx, is now mainly caused by high-risk human papillomaviruses (HPV). Indeed, HPV^+^ tonsillar cancer currently accounts for 70-80% of cases in Western Europe and the United States (1). It is more treatment sensitive and has a better prognosis than the HPV^-^ subset, which is associated with smoking of tobacco and alcohol abuse. Arguably, the difference may be explained by antigen-specific immune cell activities in the tumor microenvironment (TME) of HPV^+^ tonsillar cancer (2–5). In agreement, CD8^+^ T-cell infiltration (4, 5) and the presence of HPV-specific T-cell clones (3, 6) have been forwarded as associated with good prognosis.

The TME of solid tumors comprises a network of non-malignant cells surrounding tumor cell islets, such as fibroblasts, T-, B-, myeloid, and NK-cells. The activity of this network is of great importance for cancer progression, and much effort is invested in defining features crucial to effective immune surveillance. For example, using flow cytometry, we have recently shown a positive correlation between the frequencies of CD8^+^ T cells and dendritic cells (DC) in HPV^+^ tonsillar cancer. Furthermore, lesions with high levels of CD8 transcripts also associate with the presence of antigen-presenting cells (APC) (7), suggesting an antigen-presenting potential in the TME. However, regarding cancer cells as well as immune cells, tonsillar cancers are heterogenous lesions: therefore, such data need to be spatially resolved.

Examples where spatial resolution is needed are manyfold, such as in the context of HLA-I and HLA-II expression in HPV^+^ and HPV^-^ tonsillar cancer in relation to survival (8, 9), as well as of implications of presence/activity of regulatory T-cells (Tregs), dendritic cells (DCs), and macrophages (4, 7). Some areas have started to be explored: for example, expression of the immune checkpoint PD-L1 in the TME of tonsillar cancer is not correlated to better overall survival (OS) when evaluated in whole tissue specimens, but is associated with immune infiltration and better response to standard treatment as well as immunotherapy when expressed on macrophages and CD8^+^ T-cells infiltrating cancer-cell islets (10, 11).

Techniques available to evaluate the TME are rapidly evolving, and the use of multiplex flow cytometry and single-cell RNA sequencing have deepened the knowledge of immune cell subtypes and their transcriptomic signatures. However, these techniques cannot describe the spatial distribution of the different cells, which arguably has a potential to predict clinical outcomes, aid in selection of treatment, and define new therapeutic targets (10, 12). Recently, new platforms have entered the market directed toward the spatial analysis of tissue samples, and there are now several opportunities to overcome these limitations.

In this study, the GeoMx™ Digital Spatial Profiling platform (DSP) was used for quantitative analysis of proteins in areas of illumination (AOI) within selected regions of interest (ROI) on formalin-fixed paraffin-embedded (FFPE) tonsillar cancer tissue. We characterized CD8^+^ cells inside and outside (i.e., stroma) PanCK^+^ cancer-cell islets, and CD11c^+^ myeloid antigen-presenting cells (APC) in stroma, by immunofluorescence (IF) and DSP. We used CD8^+^ cell quantification to categorize the lesions into immune phenotypes and assessed their correlation to HPV-status and survival. An antibody panel of 43 tumor and immune cell markers was applied to investigate ROI-specific protein expression levels in AOIs.

## Materials and methods

### Sample cohort

FFPE tissue samples from 114 patients, diagnosed with tonsillar cancer in the Southern Health Care Region of Sweden in 2010-2014, were collected from the Department of Pathology at Skåne University Hospital (**Table 1**). Furthermore, four tonsils from patients subjected to elective tonsillectomy for non-malignant conditions were collected from Ängelholm Hospital. All research was performed in accordance with relevant guidelines and after approval by the Ethics Review Authority. Medical records of the tonsillar cancer patients were reviewed, and the tumors were re-classified according to the TNM classification (7^th^ edition) (13).

**Table 1:** Clinicopathological characteristics of the cohort.

	Feature	No. of patients (n=105)
**Sex**	Male/Female	76/29
**Age at diagnosis: median (range)**	60 (36–81)	
**Smoking**	NS/DS/X/U*	33/40/31/1
**HPV status**	Positive	92
	*HPV^+^/p16^+^/HPV^+^p16^+^*	*37/42/13*
	Negative	13
	*HPV^-^/p16^-^/HPV^-^p16^+^*	*7/5/1*
**T category (TNM-7)**	T1/T2/T3/T4	21/51/20/15
**Tumor stage (TNM-7)**	I/II/III/IVA/IVB/IVC	2/8/14/71/8/2
**Primary treatment and intention**	Curative	101
	RT/CRT/CRT+ND/RT+CB/RT+ND/RT+ND+S**	*74/5/1/2/18/1*
	Palliative	4
	*RT/none*	*3/1*

*NS, non-smoker; DS, daily smoker; X, ex-smoker > 1 year; U, unknown

**RT, radiotherapy; CRT, Chemoradiotherapy; ND, Neck dissection; CB, Cetuximab; S, Surgery.

**Figure 1:**
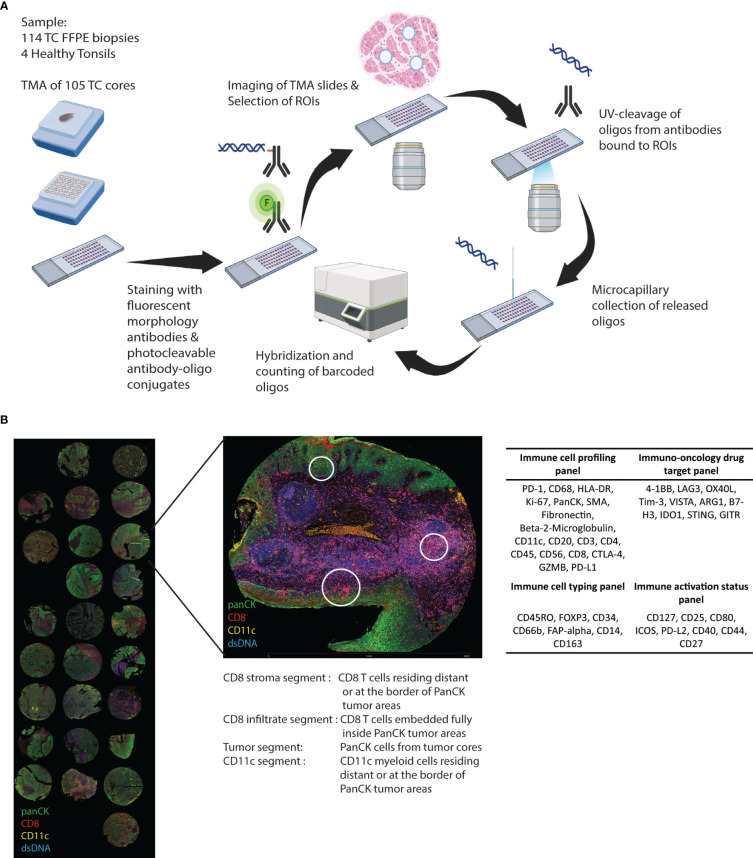
Study outline. **(A)** Cores from FFPE biopsies from 105 tonsillar cancer samples and four healthy control tonsils were transferred to TMAs and GeoMX DSP protein panel experimental procedure was performed. **(B)** The biopsy cores were morphologically assessed using DNA stain and fluorochrome-coupled antibodies towards PanCK, CD8, and CD11c. Regions of interest (ROIs) were selected inside and outside cancer cell islets. Antibodies covalently linked to an oligonucleotide UV-cleavable linker from four different profiling panels were added, and collected oligonucleotides were digitally counted using the GeoMx Digital Spatial platform.

All tumor samples were morphologically assessed using hematoxylin-eosin staining and tumor areas were marked by an experienced pathologist. Tissue microarrays (TMA) from the FFPE samples were prepared by extracting 2 mm-wide cylindrical tissue cores and transferring them to recipient blocks using the automated tissue arrayer TMA Grand Master (3DHISTECH, Budapest, Hungary) (**Figure 1A**). Assembled TMA blocks were sectioned using Microm HM355S Microtome (Thermo Scientific, Waltham, MA). Of the 114 patients, the quality of the biopsies from nine patients was considered inadequate for the analysis due to insufficient material.

### GeoMx digital spatial profiling

Glass slides with 5-µm sections of the FFPE TMAs were stained with DNA stain (SYTO™ 13 Green Fluorescent Nucleic Acid Stain, Invitrogen, Waltham, MA) and fluorescently labelled antibodies against CD8 (1:500) (CD8-AF647, clone OTI3H6, Origene, Rockville, MD), PanCK (1:1000) (pan-Cytokeratin-AF594, clone AE1+AE3, Novus Biologicals, Littleton, CO), and CD11c (1:200) (CD11c-unconjugated, clone EP1347Y, AbCam, Cambridge, UK) conjugated to AF532 with Alexa Fluor™ 532 Antibody Labelling Kit (Invitrogen) (**Figure 1A**).

Forty-three oligonucleotide-tagged antibodies from four predesigned panels (**Figure 1B**), i.e., “Immune cell profiling”, “Immunology drug targets”, “Immune activation status”, and “Immune cell typing” (Nanostring, Seattle, WA), were added to the slides. Slides were imaged and visualized using the GeoMx DSP instrument (Nanostring). DNA, PanCk, CD11c, and CD8 stainings were used to guide the selection of ROIs inside and outside (i.e., stroma) cancer-cell islets. Selected ROIs were segmented into AOI based on the location of PanCK^+^, CD8^+^, and CD11c^+^ cells (**Figure 1B**). After UV exposure, bound reagents in each AOI were collected into separate wells on a microtiter plate and quantified using a nCounter flex analysis platform (Nanostring) (**Figure 1A**).

### Quantification of CD8 cells by IF

CD8 expression was quantified using QuPath (0.3.2) (14). High-resolution images of the TMA scan taken during GeoMx DSP collection were uploaded, and a TMA grid was selected identifying each core on a TMA slide. CD8^+^ cells on each TMA core were identified and quantified using the “positive cell detection” function of QuPath. CD8^+^ cells were identified using a mean fluorescence intensity cut-off over 50 arbitrary units and a cell nuclei size cut-off over 10 pixel^2^. The area of each core on the TMA grid was calculated with QuPath and used to normalize the number of CD8^+^ cells, accounting for folded, partial, or missing cores on the TMA slide.

### Survival

Differences in survival were assessed by log-rank tests. Independence between categorical variables, such as immune markers and clinical variables, was tested using either Pearson’s Chi-square test or Fisher’s exact test (when counts were lower than five). Three multivariate Cox proportional hazards (PH) models were fitted to estimate hazard ratios for associations between variables and all-cause mortality. The models were adjusted for TNM-7 stage, HPV-status, age at diagnosis, and either total CD8^+^ cells or CD8^+^ cells outside (i.e., stroma) or inside cancer-cell islets, respectively. Survival analysis was performed using the survival and survminer R packages. The code can be made available upon reasonable request.

### GeoMx data analysis

The initial dataset including counts for each protein marker in the AOIs was processed using the GeoMx DSP Analysis Suite (Nanostring). AOIs with a minimum surface area of fewer than 1600 pixels were removed during quality assessment. The dataset was then scaled according to nuclei counts and normalized according to housekeeping proteins Ribosomal Protein S6 (S6) and Histone H3 (H3). Downstream analysis and visualization of the scaled and normalized dataset were performed using R statistical software. Principal component analysis (PCA) and heatmaps were used for data visualization using the “PCAtools” and “pheatmap” R packages, respectively.

Calculations of differentially expressed proteins between AOI locations and AOIs tied to clinical parameters were performed using Generalized linear mixed modelling (GLMM) of the data, with a patient identifier, TMA slide number, and sex used as random effects for the dataset. Differentially expressed proteins were identified using GLMM comparisons (adjusted p.value < 0.05) in the “variancePartition” R package. Volcano plots and other visualizations of differentially expressed proteins were constructed using the “ggplot2” R package.

## Results

### Patient characteristics

Clinical and pathological characteristics of the 105 patients, with a median age of 60 years and a range of 36-81, are presented in **Table 1**. One hundred-one of the 105 patients received radiotherapy with curative intention (details given in **Table 1**). Two patients with distant metastases and one with multiple comorbidities received palliative radiotherapy, and one died from concomitant disease before starting palliative radiotherapy. The follow-up time was a minimum of 5 years from the date of diagnosis for all patients, except for two who were lost to follow-up due to emigration (at 15 and 56 months follow-up, respectively). HPV-status was assessed with either detection of HPV-DNA, staining for p16, or a combination of the two. One p16^+^ biopsy was HPV-DNA^-^ and was considered HPV^-^.

### Immunophenotype and extent of CD8^+^ infiltration impact survival

First, we assessed the degree of CD8^+^ cell infiltration and its impact on survival. We quantified the number of CD8^+^ cells in each tumor tissue core, and segmented them according to their location, i.e., either inside or outside (i.e., stroma) cancer-cell islets. The mean density of CD8^+^ cells was 349 cells/mm^2^, of which 55% was in the stroma (192 cells/mm^2^) and 45% in cancer-cell islets (157 cells/mm^2^). The univariable PH models showed that total CD8^+^ cells/mm^2^ and CD8^+^ cells/mm^2^ inside cancer-cell islets as well as TNM-7 stage, HPV-status, and age at diagnosis impacted the 5-year OS (**Table 2**), while CD8^+^ cells/mm^2^ in the stroma, sex, and smoking status did not. Similarly, total CD8^+^ cell abundance and CD8^+^ inside cancer-cell islets were associated with significantly lower all-cause mortality in multivariable PH models, after adjusting for TNM-7 stage, HPV-status, and age (**Table 2**). In contrast, the abundance of CD8^+^ cells in tumor stroma was not associated with 5-year OS in either univariable or multivariable PH models (**Table 2**).

**Table 2:** Adjusted hazard ratios comparing all-cause mortality between patients according to clinical and biological markers (n=105).

	Univariable models		Multivariable model 1		Multivariable model 2		Multivariable model 3	
	HR (95% CI)	P	HR (95% CI)	P	HR (95% CI)	P	HR (95% CI)	P
TNM7	2.403 (1.422-4.060)	0.001	2.403 (1.422-4.060)	0.001	2.408 (1.436-4.039)	8.6 x 10^-4^	2.283 (1.328-3.924)	0.002
HPV status	0.373 (0.141-0.986)	0.046	0.373 (0.141-0.986)	0.046	0.314 (0.121-0.817)	0.017	0.375 (0.145-0.965)	0.042
Age	1.09 (1.033-1.161)	0.002	1.09 (1.033-1.161)	0.002	1.104 (1.041-1.171)	9.5 x 10^-4^	1.099 (1.038-1.162)	0.001
Sex	1.462 (0.592- 3.607)	0.410	–	–	–	–	–	–
Smoking status	1.295 (0.907- 1.847)	0.154	–	–	–	–	–	–
Total CD8 (cells/mm^2^)	0.997 (0.995-0.999)	0.005	0.997 (0.995-0.999)	0.038	–	–	–	–
Stroma CD8 (cells/mm^2^)	0.998 (0.995- 1.001)	0.141	–	–	0.998 (0.996- 1.001	0.307	–	–
Infiltrate CD8 (cells/mm^2^)	0.994 (0.989- 0.998)	0.008	–	–	–	–	0.995 (0.990-0.999)	0.032

-: not included in the model.

Next, we compared the effect of CD8^+^ cell abundance and location on disease-free survival (DFS) (**Table 3**). Univariable PH models showed that total and tumor-infiltrating CD8^+^ cells/mm^2^ as well as TNM7 stage, and age at diagnosis impacted 5-year DFS (**Table 3**), while CD8^+^ cells/mm^2^ in the stroma, HPV and smoking status, and sex did not. Multivariable PH models including significant variables of the univariable analyses displayed that total CD8^+^ cell/mm2 and TNM7-stage, respectively, were associated with a lower and higher risk of relapse.

**Table 3:** Adjusted hazard ratios comparing time to progression/relapse between patients according to clinical and biological markers (n=105).

	Univariable models		Multivariable model 1		Multivariable model 2		Multivariable model 3	
	HR (95% CI)	P	HR (95% CI)	P	HR (95% CI)	P	HR (95% CI)	P
TNM7	4.164 (2.176-7.971)	1.66 x 10^-5^	3.642 (1.974- 6.720)	3.51 x 10^-5^	3.766 (2.033-6.975)	2.48 x 10^-5^	3.416 (1.841-6.338)	9.74 x 10^-5^
HPV status	0.543 (0.206-1.432)	0.217	–	–	–	–	–	–
Age	1.06 (1.005-1.117)	0.031	1.043 (0.990-1.099)	0.106	1.048 (0.993-1.105)	0.088	1.051 (0.999-1.105)	0.0508
Sex	1.031 (0.452- 2.348)	0.942	–	–	–	–	–	–
Smoking status	0.666 (0.416 – 1.066)	0.090	–	–	–	–	–	–
Total CD8 (cells/mm^2^)	0.997 (0.996-0.999)	0.016	0.997 (0.995-0.999)	0.038	–	–	–	–
Stroma CD8 (cells/mm^2^)	0.998 (0.995- 1.001)	0.156	–	–	0.998 (0.995- 1.001	0.156	–	–
Infiltrate CD8 (cells/mm^2^)	0.996 (0.992- 0.999)	0.031	–	–	–	–	0.996 (0.993-1.000)	0.076

-: not included in the model.

**Figure 2:**
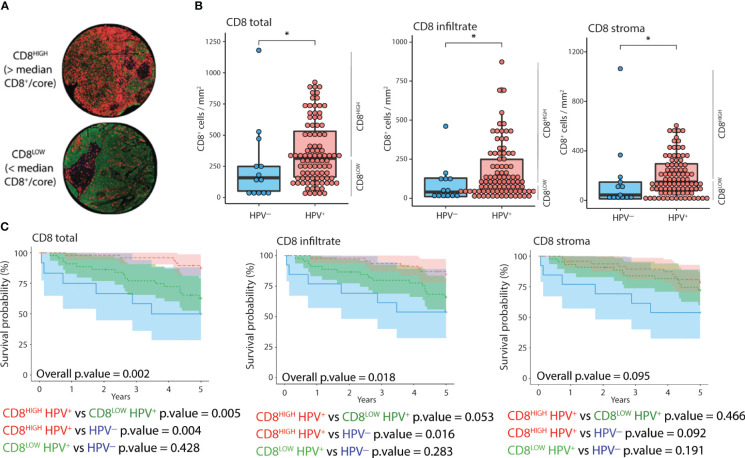
Distribution and impact of CD8 and HPV status on 5-year OS. **(A)** IF images of two representative cores corresponding to CD8^HIGH^ and CD8^LOW^ tonsillar cancer tumors (red: CD8, green: panCK, yellow: CD11c, blue: DNA). **(B)** Normalized counts of CD8^+^ cells according to HPV status in different tumor compartments, i.e., overall as well as inside and outside cancer-cell islets. **(C)** Kaplan-Meier curves displaying 5-year OS (n=105), stratified according to HPV and CD8 status in different tumor compartments. CD8^HIGH^ and CD8^LOW^ HPV^+^ tumors refer to the groups of patients with CD8 expression above or below the median value. The Y axis represents years since diagnosis. *: p.value < 0.05.

To assess the prognostic value of combining CD8 quantification and HPV status, we stratified patients according to both parameters (**Figure 2**). We classified the patients into CD8^HIGH^ and CD8^LOW^ tumors according to the median number of CD8^+^ cells (**Figure 2A**). HPV^+^ tumors displayed significantly higher CD8^+^ cell counts in the stroma, cancer-cell islets, and overall core surface compared to HPV^-^ tumors (**Figure 2B**). Patients featuring CD8^HIGH^ HPV^+^ tumors had significantly lower all-cause mortality than patients with CD8^LOW^ HPV^+^ tumors and HPV^-^ tumors, respectively, when total CD8^+^ cell counts were considered (**Figure 2C**, left-most Kaplan-Meier curve). Similar trends were observed when comparing all-cause mortality in the three groups with the level of CD8 infiltration, but only the comparison between CD8^HIGH^ HPV^+^ and HPV- reached statistical significance. No statistically significant differences in all-cause mortality were observed when grouping tonsillar cancer patients according to HPV status and CD8^+^ cell abundance in stroma or cancer cell islets (**Figure 2C** middle and right-most Kaplan-Meier curves respectively).

### Location-segmentation of CD8^+^ and CD11c^+^ cells and the effect of TME on immune cells

**Figure 3:**
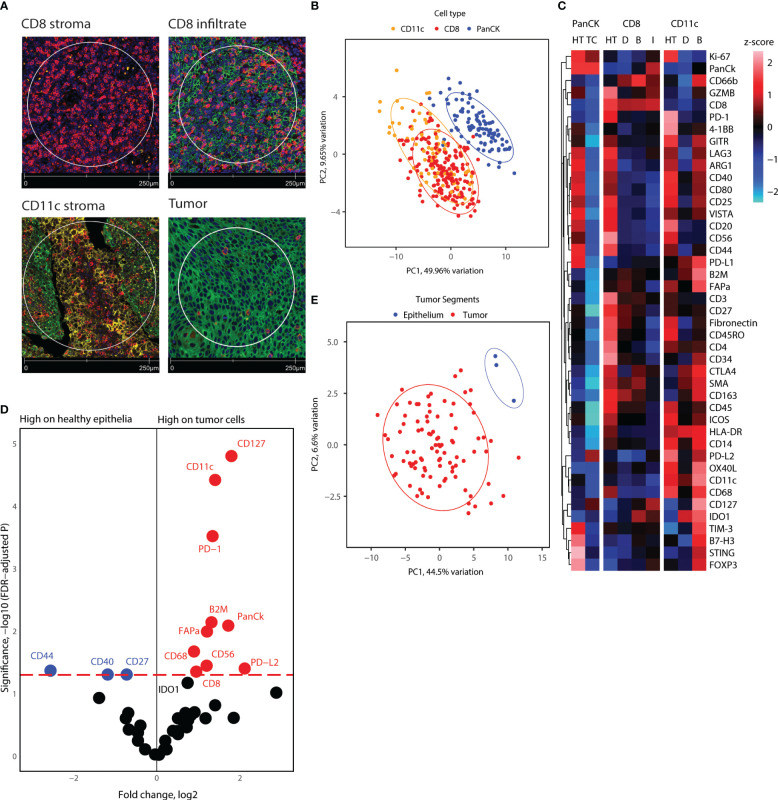
Overview of proteome for all segments collected, and tumor cell and healthy epithelium proteome comparison. **(A)** Representative immunofluorescence staining pictures of the segment location strategy and cell types. **(B)** PCA distribution of all segments in tonsillar cancer and healthy tonsil tissue. **(C)** Heatmap showing scaled protein counts for all segment types in the dataset, separated for healthy tonsil and tonsillar cancer origin, epithelial, CD8, and CD11c segments, and the locations the segments were extracted from. Annotation abbreviations are HT, Healthy tonsil; tonsillar cancer, Tonsillar cancer; D, Distant; B, Tumor boundary; I, Infiltrate. **(D)** Volcano plot showing significantly different protein counts between epithelial cells between tonsillar cancer biopsies and healthy tonsils. Significance threshold FDR< 0.05. **(E)** PCA distribution of epithelial segments in tonsillar cancer and healthy tonsil tissue.

Using Nanostring GeoMX™ DSP, we evaluated the expression of a 43-protein panel across selected AOIs using DSP. 355 AOIs were selected across four TMA sections based on the expression of CD8, CD11c, and PanCK, as assessed by IF. We defined four types of ROIs, including areas enriched in CD8^+^ cells located in stroma, CD8^+^ cells inside cancer-cell islets, CD11c^+^ cells located in the stroma, and panCK^+^ regions (**Figure 3**). To obtain an overview of the DSP protein dataset, we performed PCA across AOIs, followed by visualization of protein expression on CD8^+^, CD11c^+^, and panCK^+^ AOIs selected from tumor cores (**Figures 3B, C**). Cancer cell AOIs expressed high levels of panCK, Ki-67, and PD-L2, and were proteomically distinct from both CD8^+^ and CD11c^+^ AOIs. In turn, CD8^+^ AOIs featured high levels of CD8, CD3, and CD45, and displayed a certain degree of divergence from CD11c^+^ AOIs. CD11c^+^ segments showed higher expression of most markers included in the protein panel, except panCK and Ki-67 (**Figure 3C**). Together, these results indicate differences in protein expression between the cell types studied, as well as a successful enrichment of canonical proteins in PanCK and CD8 AOIs, validating the segmentation strategy. CD11c^+^ AOIs, due to the size of the cells and their proximity to other immune cells and niches were found to contain non-myeloid cell-related markers such as CD20, PD-1, CD4, and CD3 to an extent.

### Epithelial cells of healthy tonsils and tonsillar cancer (cancer cells) are proteomically different

Next, we investigated the differential protein expression between panCK^+^ epithelial cells from healthy tonsils and malignant cells from tonsillar cancer biopsies. Because adjacent protein expression can be obtained through DSP, we hypothesized that comparison of epithelial cell segments could yield information on the receptor-receptor interactions and cell niches that reside inside tumor cell islets. PCA analysis of the healthy tonsil epithelial cells and all tumor segments from tonsillar cancer patients indicated proteomic divergence between healthy and malignant cells (**Figure 3E**). We employed generalized linear mixed modelling (GLMM) of the DSP dataset to account for discrepancies in the dataset such as multiple segments of the same kind from each tumor core and batch effects from tumor cores on different microarray slides. GLMM differential protein expression indicated immune checkpoint markers such as PD-1, PD-L1, PD-L2 with elevated expression on tumor cells and nearby immune niches. Tumor cell segments also display higher expression of CD8, CD11c, CD56, CD68, and CD127, indicating an enriched immune infiltration comprising of cytotoxic T-cells, antigen-presenting cells such as DCs and macrophages near tumor cell segments (**Figures 3C, D**). On the other hand, tumor cell segments displayed lower levels of CD44, CD40, and CD27 (**Figure 3D**). Overall, tumor cell islets underline an enriched immune cell compartment, but give indications of immune cell suppression and engagement of immune checkpoint receptors.

### Checkpoint and co-stimulatory markers are elevated on CD8^+^ T-cells in proximity to tumor cell islets

**Figure 4:**
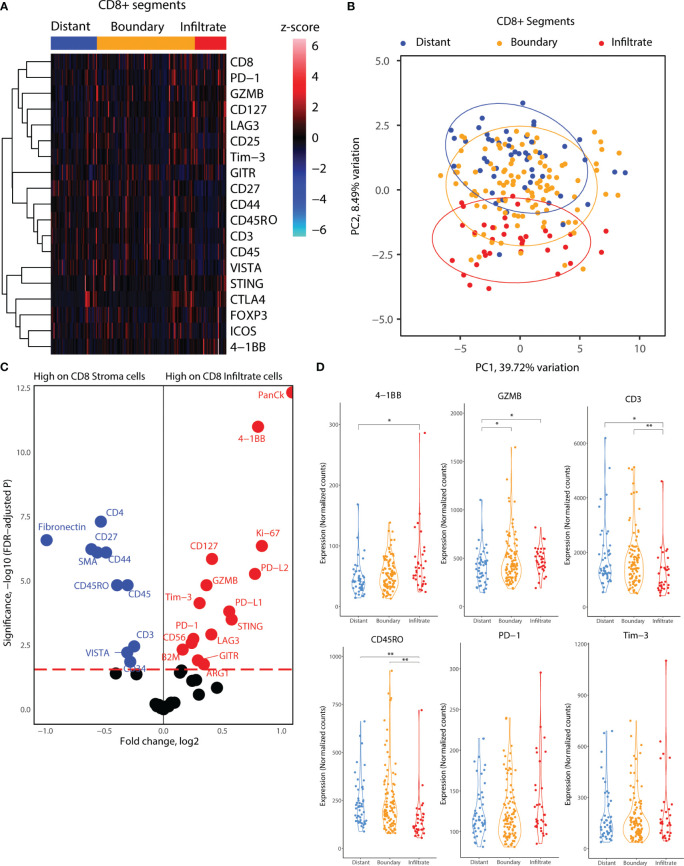
Location-based proteome comparisons for CD8 segments collected from tonsillar cancer biopsies. **(A)** Heatmap showing scaled protein counts for CD8-related proteins between distant and tumor boundary CD8 cell clusters and infiltrated T cells. **(B)** PCA distribution of CD8 segments according to their location in tumor biopsies and 43 protein markers assessed. **(C)** Volcano plot showing significantly different protein counts between CD8 cells of different locations in tonsillar cancer biopsies. CD8 stroma classification was taken as the segments outside tumor tissue, and CD8 infiltrate inside tumor tissue. Significance threshold FDR< 0.05. **(D)** Violin plots demonstrating functional and immune checkpoint-related markers among CD8 segments of different locations. *: p.value < 0.05, ** p.value < 0.01.

Next, we delved into the proteomic differences between CD8^+^ cells infiltrating cancer-cell islets and those located in the stroma (**Figure 4**). CD8^+^ AOIs were selected according to a location-based strategy, with CD8 infiltrate segments selected from T-cells completely inside tumor cell islets, CD8 boundary segments selected from T-cells in contact or in close proximity to tumor-stroma boundary, and CD8 distant segments consisting of T-cell clusters not in contact with tumor cell islets. Tonsillar cancer biopsies with a deserted immunophenotype, or extensive tumor cell presence, prevented the selection of one or more CD8 segment types for each tonsillar cancer biopsy; accordingly, the representation of each AOI in the dataset is not equal. Scaled visualization of T-cell-related markers showed expression variation of principal markers such as CD3, CD45, CD45RO, and CD27 between infiltrate and stroma T-cell AOIs (**Figure 4A**). PCA overview of the CD8 segments showed considerable variation in the protein expression patterns between stroma, tumor border, and tumor infiltrates, with shifting variation according to proximity to the tumor cell boundary (**Figure 4B**). We performed location-based differential expression analysis on CD8 AOIs using GLMM. CD8^+^ cells infiltrating cancer-cell islets featured higher expression of proteins related to effector function (GZMB, CD127), T-cell co-stimulation and co-inhibition (PD-1, LAG3, 4-1BB, GITR, STING, B7-H3), and other non-T-cell co-inhibitory proteins (PD-L1, PD-L2), IDO1, and Ki-67. On the other hand, CD8^+^ cells in stroma AOIs featured higher levels of T-cell memory markers, (CD45RO, CD44), early T-cell stimulation (CD3, CD27, CD44) as well as non-CD8^+^ T-cell markers (CD4, Fibronectin, Smooth muscle actin: SMA) (**Figure 4C**). From these markers, the expression level of PD-L1 in all CD8^+^ regions was significantly associated with higher and lower all cause-mortality when tested in univariate PH models (p.value 0.049 and 0.041). However, PD-L1 expression failed to reach statistical significance when tested in combination with multivariate PH models described in **Table 4**. Although not significant with linear mixed modelling of the data, distant and boundary CD8 T-cell ROIs showed differential expression as well. GZMB was expressed in higher levels in boundary cells compared to both infiltrated CD8 and distant CD8 T-cell clusters. Immune checkpoint markers such as PD-1, 4-1BB, and Tim-3 also showed sequential expression elevated with closer proximity to tumor cell islets, whereas CD3 showed lowered expression in infiltrated and boundary T-cells (**Figure 4D**).

**Table 4:** Adjusted hazard ratios comparing all-cause mortality for patients with tonsillar cancer according to total expression of differentially expressed proteins between CD8 AOIs outside (i.e., stroma) and inside cancer-cell islets (adjusted p.value < 0.05, FC > 1.5) (n=95) in CD8^+^ regions.

	Univariable models		Multivariable model 1 (**Table 2**)	
	HR (95% CI)	P	HR (95% CI)	P
**Fibronectin**	1.000 (0.996 - 1.004)	0.090	–	–
**CD4**	0.999 (0.999 - 1.000)	0.576	–	–
**CD27**	0.999 (0.996 - 1.002)	0.451	–	–
**SMA**	0.999 (0.998 - 1.000)	0.103	–	–
**4-1BB**	0.996 (0.989 - 1.003)	0.263	–	–
**Ki-67**	0.999 (0.999 - 1.000)	0.387	–	–
**PD-L2**	0.988 (0.974 – 1.002)	0.097	0.987 (0.975 – 1.000)	0.055
**STING**	0.999 (0.999 – 1.000)	0.274	–	–
**PD-L1**	**0.994 (0.988 - 0.999)**	**0.040**	0.996 (0.991 – 1.002)	0.219

Significant markers are indicated in bold.

### CD11c^+^ myeloid APCs display elevated levels of immune suppressor markers on the tumor-stroma boundary

**Figure 5:**
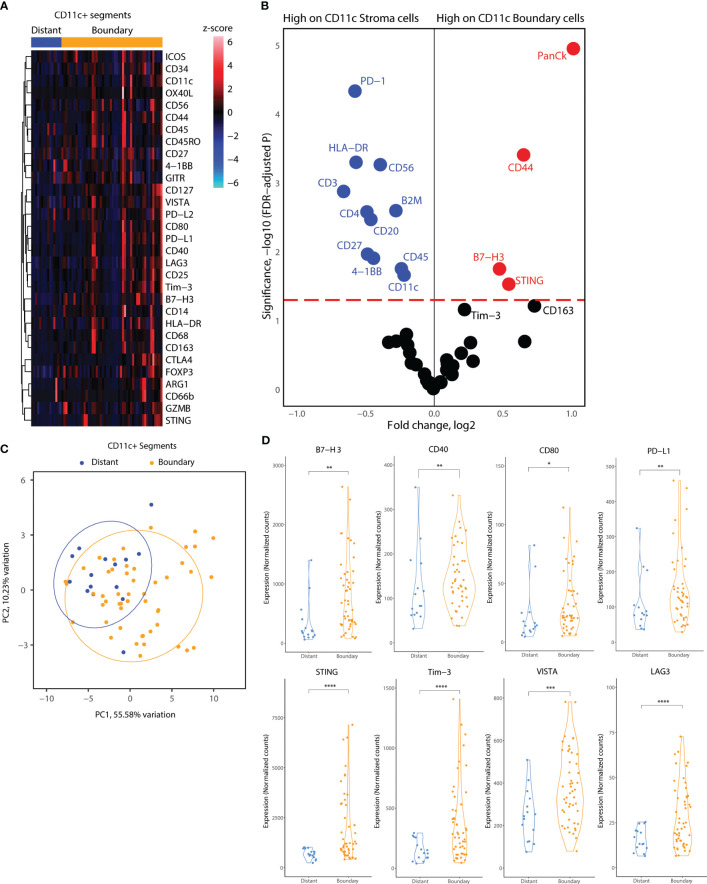
Location-based proteome comparisons for CD11c segments collected from tonsillar cancer biopsies. **(A)** Heatmap showing scaled protein counts for CD11c related proteins between distant and tumor boundary CD11c cell clusters **(B)** Volcano plot showing significantly different protein counts between CD11c cells of different locations in tonsillar cancer biopsies. Significance threshold FDR< 0.05. **(C)** PCA distribution of CD11c segments according to their location in tumor biopsies and 43 protein markers assessed. **(D)** Violin plots demonstrating functional and immune checkpoint-related markers among CD11C segments of different locations. *: p.value < 0.05, ** p.value < 0.01, ***p.value < 0.001, ****p.value < 0.0001.

Subsequently, we focused on the myeloid cell AOIs in our DSP dataset. We employed a similar location-based strategy for CD11c^+^ AOIs, consisting of CD11c^+^ distant segments, which reside away from tumor cell islets, and CD11c^+^ boundary segments, which reside at the tumor-stroma boundary. In contrast, CD11c^+^ cell clusters infiltrating tumor cell islets fully were very rare, and of insufficient cell number to collect as a segment. Heatmap visualization of all CD11c^+^ segments indicated elevated levels of co-stimulatory molecules (ICOS, OX40L, CD40, CD80) and inhibitory molecules (PD-L1, PD-L2, Tim-3, LAG3, Foxp3, CTLA4) as well as B7-H3, STING, and CD44 (**Figure 5A**). GLMM differential expression analysis of the boundary and distant CD11c^+^ segments revealed significantly changing markers according to cell location. Inhibitory molecules such as B7-H3 and Tim3 were elevated in tumor boundary CD11c cells as well as molecules related to interferon production such as STING. CD11c^+^ cell clusters residing away from the tumor boundary seemed to incorporate many immune cell niches in their environment, indicated by the expression of CD3, CD4, CD45, CD56, CD20, and CD27 in CD11c^+^ distant segments. Interestingly, lower expression of HLA-DR was observed on CD11c^+^ cells in tumor boundary as compared to distant segments (**Figure 5B**). PCA according to all molecular markers revealed that CD11c^+^ cells were proteomically divergent based on location in the TME (**Figure 5C**). Expression levels of B7-H3, PD-L1, Tim-3, LAG3, VISTA, and STING as well as of CD40 and CD80 were significantly elevated in tumor boundary CD11c^+^ segments (**Figure 5D**).

### CD8^+^ T cells and CD11c^+^ in tonsillar cancer have an enriched immune marker milieu compared to immune cells in healthy tonsils

**Figure 6:**
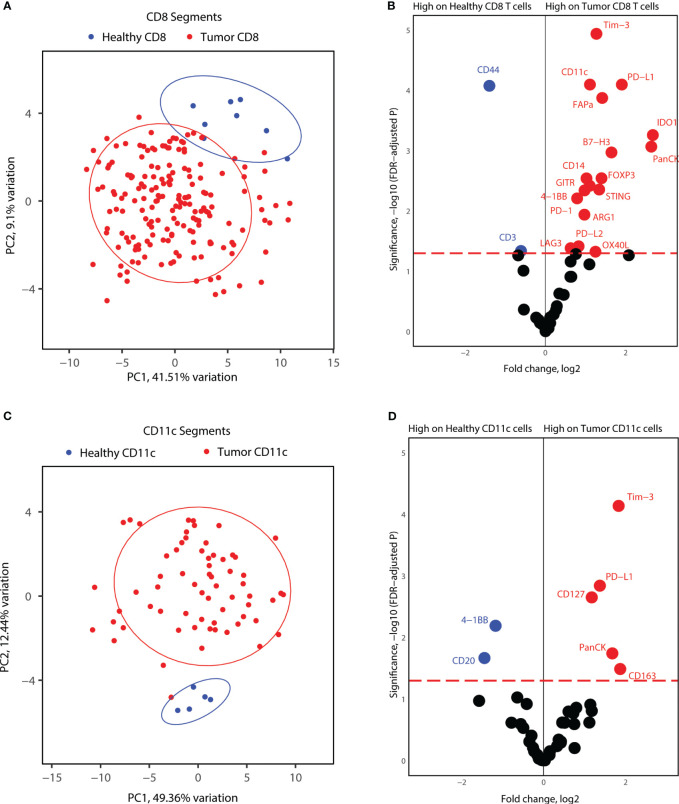
Proteome comparisons for CD11c and CD8 segments collected from tonsillar cancer biopsies and healthy tonsils. **(A)** PCA distribution of CD8 segments in tonsillar cancer and healthy tonsil segments. **(B)** Volcano plot showing significantly different protein counts between CD8 cells between tonsillar cancer biopsies and healthy tonsils. Significance threshold FDR< 0.05. **(C)** PCA distribution of CD11c segments in tonsillar cancer and healthy tonsil segments. **(D)** Volcano plot showing significantly different protein counts between CD11c cells between tonsillar cancer biopsies and healthy tonsils. Significance threshold FDR< 0.05.

Lastly, we investigated whether tumor CD8^+^ T-cells and CD11c^+^ cells express unique markers compared to immune cells from healthy tonsils. For this analysis, we considered the segments for each cell type together, as the number of healthy tonsils AOIs was not sufficient to include different locations in each biopsy. PCA analysis of CD8^+^ segments indicated differences in the protein expression profiles of differences between healthy and tumor CD8^+^ T-cells (**Figure 6A**). Differential protein expression analysis between healthy tonsils and tumor CD8^+^ T-cell segments revealed higher expression of many immune markers in tumor CD8^+^ T-cells. Immune checkpoint molecules (PD-1, PD-L1, PD-L2) and immune suppressor markers (Tim-3, FOXP3, LAG3) were all significantly expressed on tumor T-cells as well as co-stimulatory molecules (STING, B7-H3, OX40L, 4-1BB). Although many differentially elevated markers were not specific to cytotoxic T-cells, these might represent segment collection from other immune cells with receptor-receptor interactions in proximity to T-cell clusters (**Figure 6B**). We also performed the same analysis for healthy and tumor CD11c^+^ segments. Similar to CD8^+^ T-cell segments, healthy tonsil and tumor CD11c^+^ cell segments showed variance according to disease status (**Figure 6C**). Differential expression analysis revealed significantly elevated protein expression for PD-L1 and Tim-3 in tumor segments as well as for CD127 and CD163. 4-1BB; an activation-induced co-stimulatory molecule, was found to be less expressed in tumor CD11c^+^ segments (**Figure 6D**). Overall, the results highlight an overexpression of immune checkpoint and immune suppressor molecules by both APCs and cytotoxic T-cells in the tumor microenvironment.

## Discussion

Immune cell activity in the TME is determined by many parameters, including the immune cell composition, proximity to tumor tissue, contact points of different immune cells to each other and to malignant cells, and the expression of individual immune receptors on cells (6). Observational and functional studies conducted on immune cells by flow cytometry or single-cell sequencing lose this crucial spatial information due to the requirement of tissue processing and single-cell suspensions. Therefore, spatial analysis of the TME will be indispensable for understanding cellular and molecular interactions within the tumor. Immune checkpoint inhibitors, such as anti-PD-1, anti-PDL1, and anti-CTLA4 have been approved as treatment options for HNSCC, but the response rate is only 10-20% (15). Spatial proteomics and transcriptomics have the potential to show us how the expression patterns for immunotherapy targets change across patients, cells, and tumor tissue.

In this study, we used the Nanostring GeoMx Digital Spatial Profiler (DSP) to describe and characterize tumor location-based differences in immune cell markers and to compare healthy tissue and tonsillar cancer biopsies. We devised an AOI selection strategy based on having types of segments with increasing proximity to tumor cell islets and focused our analysis on two important components of the TME: cytotoxic T-cells and antigen-presenting cells of myeloid origin, which include DCs and macrophages. We found that various immune checkpoint molecules and immune suppressor molecules increased protein expression with closer proximity to TME. Strikingly, this pattern was also repeated for costimulation-related markers as well, granting a two-sided overview to the dataset (**Figure 3C**).

We first connected the state of the immune landscape of tonsillar cancer tumors to clinical parameters. Immune phenotyping based on CD8^+^ T-cell biology has been related to patient prognosis in HPV^+^ tonsillar cancer. Nordfors et al. highlighted that the degree of CD8^+^ infiltration positively correlated with an increase in 3-year OS in tonsillar cancer (5). In this study, we observed that the total abundance of CD8^+^ cell infiltration was associated with higher 5-year OS and DFS, after correcting for TNM7 tumor stage, age at diagnosis, and HPV status (**Table 2**). In addition, HPV^+^ tonsillar cancer displayed a higher degree of CD8 T-cell abundance across tumor cores compared to HPV^-^ tonsillar cancer. Upon segmentation of CD8 abundance in stroma and cancer cell islet regions, we observed a similar pattern, where HPV^+^ tonsillar cancer featured higher CD8 abundance compared to HPV^-^ lesions. Notably, the relative abundance of CD8^+^ cells infiltrating cancer-cell islets correlated with improved patient prognosis and specifically in HPV^+^ tonsillar cancer, while the relative abundance of CD8^+^ cells in the stroma did not (**Figure 2**, **Table 2**). Conversely, Oguejiofor et al. reported that only the relative abundance of CD8^+^ cells in the stroma were correlated with higher 5-year OS in HPV^+^ oropharyngeal cancer patients (16). The discordance between both studies might be caused by differences in tumor location and the immune context of each tissue type in the head and neck area. While we described events specifically in tonsillar cancer patients, Oguejiofor et al. used a cohort of oropharyngeal cancer patients, including base of the tongue and other oropharyngeal cancer subsites. Our results suggest the degree of CD8^+^ cell infiltration in cancer-cell islets as a prognostic biomarker, which may be used in the clinical setting during tonsillar cancer patient stratification and treatment selection.

Studies performed on head and neck cancer biopsies utilizing the spatial architecture of the tumor have identified markers such as PD-L1, PD-1, STING, IDO1, GZMB, and Ki67 to be differentially expressed between different locations of the tumor and being predictive of progressive disease (17). Kulasinghe et al. utilized a proof-of-concept approach by sampling tumor infiltrating edge, immune-only, and tumor-only areas from whole biopsies in a small patient cohort, indicating transcriptomic differences of immune cells inside and outside tumor islets. Recent studies from the same group integrated spatial expression of immune markers with response to therapy in HNSCC patients: 4-1BB expression in the tumor compartment, but not in the stroma, was associated with superior overall survival. Similarly, high CD27 expression in the stroma was found to be associated with better survival outcomes (18).

In our study, we showed a sequential increase in protein expression for PD-1, PD-L1, and PD-L2 from healthy to malignant tissue, and subsequently from distant stroma to tumor border and infiltrated cells (**Figure 4C**). In the TME, tumor cells exploit cell exhaustion and cellular inactivation processes by expressing PD-L1 and PD-L2 themselves and inducing PD-1 on cytotoxic T-cells and PD-L1/PD-L2 on APCs (19). Increased expression of these immune checkpoint molecules in cells closest to the tumor cell islets support an effect of the TME on re-programming of the immune system to avoid cancer cell recognition. We also identified B7-H3 as having elevated protein expression in tumor CD8^+^ T cells and tumor boundary CD11c^+^ cells (**Figures 5B**, **6B**). B7-H3 expression is linked to immune suppression, and solid tumors such as ovarian, gastric, brain, and breast tumors are shown to highly express B7-H3 in correlation with poor prognosis and increased tumor size (20). In concordance with the expression patterns of immune checkpoint molecules, we demonstrated increased expression of immune suppressor markers such as FOXP3, Tim-3, and LAG3 on tumor CD8 T cells and CD11c^+^ cells with proximity to tumor tissue (**Figures 4C**, **6B**). The TME is known to induce Treg differentiation on infiltrating T-cells, essential for maintaining immune tolerance (21). Interestingly, CTLA4 expression was stable across healthy and tumor tissue, and in the different locations of tumor biopsies, which might be a point of strength for its potential as a therapeutic target for immunotherapy.

Another interesting observation we made was the significant increase in 4-1BB expression in tumor-infiltrating CD8^+^ T-cells in comparison to stromal T-cells (**Figure 4C**). 4-1BB is a cell surface glycoprotein and functions as a costimulatory receptor, initiator of cytokine secretion, and maintenance of effector T-cell functions (2). Agonistic targeting of 4-1BB in cancer immunotherapies has been shown to induce tumor regression and activation of the immune system (22). We postulate that 4-1BB protein expression may be a predictive marker for the state of the tumor environment, being elevated specifically on tumor infiltrating T cells. Imaging of 4-1BB expressing cells or expression based analysis of bulk tumor protein or RNA will allow us to classify the infiltration state of the tumor, and assess response to therapy as well as, predict patient specific therapy strategies.

In our study, the increase in expression of co-stimulatory molecules on tumor boundary was even more pronounced in CD11c^+^ cells in tonsillar cancer. We showed an increase in expression for a wide range of co-stimulatory molecules, CD40, CD80, OX40L, and STING on tumor boundary CD11c^+^ cells (**Figure 5D**). This “double-edge” observation of both co-stimulation molecules and immune suppressor markers, featuring increased expression with proximity to tumor cell islets, might suggest that immune cells are primed for effector functions, but are kept suppressed by the TME. Therefore, targeting these molecules together, antagonistic for immune checkpoint, and agonistic for co-stimulatory factors, may have a synergistic effect in cancer immunotherapy. Indeed, recent studies have shown that combination of anti-4-1BB and anti-CTLA4 have a cumulative effect on T-cell activation, tumor rejection, and patient survival (23). Anti-4-1BB has also been used in combination with anti-PD-1 and radiotherapy for better outcomes of tumor immunotherapy (24).

In addition to functional markers, we observed that molecules related to T-cell memory, i.e., CD45RO and CD27, were downregulated on tumor-infiltrating T-cells and were expressed predominantly in stromal T-cells, which may indicate that tissue resident-memory T-cells are confined to the tumor stroma and are unable to contact tumor cells (**Figure 4C**). An extensive or full transcriptomic panel of markers is necessary to understand the state of memory T-cells in tonsillar cancer, and their function according to the localization of these cells.

During the analysis of our data set, we observed that several cell type-specific proteins were detected on other cell types analyzed (e.g., PanCK and CD4 expression on CD8^+^ segments), highlighting a limited specificity of DSP. We postulate that the detection of non-cell-specific proteins from AOIs represents either the immediate vicinity of the cells being analyzed or the specificity of the marker used in IF. For instance, PanCK, PD-L1, and PD-L2 detection in CD8^+^ cells infiltrating cancer-cell islets may be a carryover of material from tumor cells in proximity. On the other hand, expression of CD20 by CD11c^+^ cells may represent intra-tumoral infiltration of rare CD11c^+^ B-cells (25, 26). Keeping these observations in mind, we drew our conclusions from protein markers that were specific for each immune cell type selected in our AOIs.

In light of all our observations, we also would like to highlight some limitations of data analysis and the assay platform we used. Spatial proteomics of tissue sections is still an emerging field, and new technologies and optimizations of existing platforms are being made consistently. GeoMX DSP platform utilizes selection of a fluorescent marker-based AOI inside of a broader region of interest, and thus collects barcodes from a cluster of target cells rather than single cell or sub-cellular precision. We aimed to keep our AOI collection area from each region of interest segment >50, to ensure a similar number of barcodes will be collected from each core across the TMA, and subsequent batches. Moreover, we normalized our dataset not only according to housekeeping proteins Ribosomal protein (S6) and Histone H3, but also with nucleus count of each AOI, assessed by DAPI staining obtained during imaging, ensuring that small and large AOI were comparable. Moreover, the barcoded antibodies that were used in the GeoMX DSP assay panels were chosen and conjugated by Nanostring and assessed for staining and binding efficiency. During our data analysis, we postulated that the amount of barcodes collected from each AOI would correspond to the amount of antibody binding to the target, and would reflect the amount of target protein in the area of interest. Lastly, another issue would be the representation of the tumor microenvironment. Construction of the TMA takes only a small 2mm core from the biopsy obtained, which is also a small portion of a larger tumor present in the patient. Furthermore, we selected ROI and AOI from these TMA cores, which is a further sub-sampling of the tumor itself. Thus, the core and the further selections may not be representative of the tumor as a whole. To minimize this selection bias, we obtained expert opinion from our pathologists, and marked each biopsy with a core extraction area that would be a representation of the general architecture of the biopsy tissue.

To conclude, we analyzed both CD8^+^ T cells and CD11c^+^ cells in tonsillar cancer tumors and found location-based differences in immune checkpoint molecules and co-stimulatory markers on both immune cell types. We also observed that there was a positive association between CD8^+^ cell infiltration in cancer-cell islets and 5-year OS in tonsillar cancer (independent of HPV-status), which may be related to an effector CD8^+^ T-cell profile in cancer-cell islets. Moreover, 4-1BB protein expression showed the most elevated change between stromal and infiltrated CD8^+^ T cells, highlighting a possibility to utilize 4-1BB expression as a classifier for tumor immune state, and as a target for immune therapy. Spatial studies, both in terms of absolute protein or RNA expression as well as image-based, will be crucial to uncover cell subsets and their functional properties in tonsillar cancer and cancers in general, and will be a crucial supplement to single-cell and *in vitro* functional analyses. We will seek to expand our knowledge base in tonsillar cancer microenvironment by conducting whole transcriptome spatial analysis, as well as subcellular location analysis of immune markers. Longitudinal spatial studies will also become more feasible, giving information on the changes in tumor architecture due to therapy and over time. Together such data may be used for patient stratification, including in the context of cancer immunotherapy, and for identification of new treatment targets.

## Data availability statement

The original contributions presented in the study are included in the article/supplementary materials, further inquiries can be directed to the corresponding authors.

## Ethics statement

This study has Human Research Ethics (HREC) approval from the Swedish Ethical Review Authority with ref. no. 2019-05071. The participants that contributed with healthy tonsil tissue provided their written informed consent to participate in this study. The studies were conducted in accordance with the local legislation and institutional requirements. The participants provided their written informed consent to participate in this study.

## Author contributions

Idea concept, ML, CA, and LG. Methodology/experimental, CA, DJ, and AS. Data analysis, CA, DJ, DA and AS. Writing and critical review, all authors. All authors contributed to the article and approved the submitted.

## References

[1] O'Sullivan B Huang SH Su J Garden AS Sturgis EM Dahlstrom K. Development and validation of a staging system for HPV-related oropharyngeal cancer by the International Collaboration on Oropharyngeal cancer Network for Staging (ICON-S): a multicentre cohort study. Lancet Oncol (2016) 17(4):440–51. doi: 10.1016/S1470-2045(15)00560-4 26936027

[2] Ang KK Harris J Wheeler R Weber R Rosenthal DI Nguyen-Tan PF. Human papillomavirus and survival of patients with oropharyngeal cancer. N Engl J Med (2010) 363(1):24–35. doi: 10.1056/NEJMoa0912217 20530316 PMC2943767

[3] Welters MJP Ma W Santegoets S Goedemans R Ehsan I Jordanova ES. Intratumoral HPV16-specific T cells constitute a type I-oriented tumor microenvironment to improve survival in HPV16-driven oropharyngeal cancer. Clin Cancer Res (2018) 24(3):634–47. doi: 10.1158/1078-0432.CCR-17-2140 29018052

[4] Nasman A Romanitan M Nordfors C Grun N Johansson H Hammarstedt L. Tumor infiltrating CD8+ and Foxp3+ lymphocytes correlate to clinical outcome and human papillomavirus (HPV) status in tonsillar cancer. PloS One (2012) 7(6):e38711. doi: 10.1371/journal.pone.0038711 22701698 PMC3373553

[5] Nordfors C Grun N Tertipis N Ahrlund-Richter A Haeggblom L Sivars L. CD8+ and CD4+ tumour infiltrating lymphocytes in relation to human papillomavirus status and clinical outcome in tonsillar and base of tongue squamous cell carcinoma. Eur J Cancer. (2013) 49(11):2522–30. doi: 10.1016/j.ejca.2013.03.019 23571147

[6] Bhatt KH Neller MA Srihari S Crooks P Lekieffre L Aftab BT. Profiling HPV-16-specific T cell responses reveals broad antigen reactivities in oropharyngeal cancer patients. J Exp Med (2020) 217(10). doi: 10.1084/jem.20200389 PMC7537390 32716518

[7] Jimenez DG Sobti A Askmyr D Sakellariou C Santos SC Swoboda S. Tonsillar cancer with high CD8(+) T-cell infiltration features increased levels of dendritic cells and transcriptional regulation associated with an inflamed tumor microenvironment. Cancers (Basel) (2021) 13(21):5341. doi: 10.3390/cancers13215341 PMC8582523 34771506

[8] Cioni B Jordanova ES Hooijberg E van der Linden R de Menezes RX Tan K. HLA class II expression on tumor cells and low numbers of tumor-associated macrophages predict clinical outcome in oropharyngeal cancer. Head Neck. (2019) 41(2):463–78. doi: 10.1002/hed.25442 PMC6519285 30549362

[9] Nasman A Andersson E Nordfors C Grun N Johansson H Munck-Wikland E. MHC class I expression in HPV positive and negative tonsillar squamous cell carcinoma in correlation to clinical outcome. Int J Cancer. (2013) 132(1):72–81. doi: 10.1002/ijc.27635 22592660

[10] Sato F Ono T Kawahara A Kawaguchi T Tanaka H Shimamatsu K. Prognostic impact of p16 and PD-L1 expression in patients with oropharyngeal squamous cell carcinoma receiving a definitive treatment. J Clin Pathol (2019) 72(8):542–9. doi: 10.1136/jclinpath-2019-205818 PMC6678043 31113825

[11] Young RJ Bressel M Porceddu S Cernelc J Savas P Liu H. Validation and characterisation of prognostically significant PD-L1(+) immune cells in HPV+ oropharyngeal squamous cell carcinoma. Oral Oncol (2020) 101:104516. doi: 10.1016/j.oraloncology.2019.104516 31838340

[12] Galon J Pages F Marincola FM Angell HK Thurin M Lugli A. Cancer classification using the Immunoscore: a worldwide task force. J Transl Med (2012) 10:205. doi: 10.1186/1479-5876-10-205 23034130 PMC3554496

[13] Edge SB Compton CC. The American Joint Committee on Cancer: the 7th edition of the AJCC cancer staging manual and the future of TNM. Ann Surg Oncol (2010) 17(6):1471–4. doi: 10.1245/s10434-010-0985-4 20180029

[14] Bankhead P Loughrey MB Fernandez JA Dombrowski Y McArt DG Dunne PD. QuPath: Open source software for digital pathology image analysis. Sci Rep (2017) 7(1):16878. doi: 10.1038/s41598-017-17204-5 29203879 PMC5715110

[15] Tosi A Parisatto B Menegaldo A Spinato G Guido M Del Mistro A. The immune microenvironment of HPV-positive and HPV-negative oropharyngeal squamous cell carcinoma: a multiparametric quantitative and spatial analysis unveils a rationale to target treatment-naive tumors with immune checkpoint inhibitors. J Exp Clin Cancer Res (2022) 41(1):279. doi: 10.1186/s13046-022-02481-4 36123711 PMC9487049

[16] Oguejiofor K Hall J Slater C Betts G Hall G Slevin N. Stromal infiltration of CD8 T cells is associated with improved clinical outcome in HPV-positive oropharyngeal squamous carcinoma. Br J Cancer. (2015) 113(6):886–93. doi: 10.1038/bjc.2015.277 PMC4578081 26313665

[17] Kulasinghe A Taheri T O'Byrne K Hughes BGM Kenny L Punyadeera C. Highly multiplexed digital spatial profiling of the tumor microenvironment of head and neck squamous cell carcinoma patients. Front Oncol (2020) 10:607349. doi: 10.3389/fonc.2020.607349 33542903 PMC7851078

[18] Sadeghirad H Liu N Monkman J Ma N Cheikh BB Jhaveri N. Compartmentalized spatial profiling of the tumor microenvironment in head and neck squamous cell carcinoma identifies immune checkpoint molecules and tumor necrosis factor receptor superfamily members as biomarkers of response to immunotherapy. Front Immunol (2023) 14. doi: 10.3389/fimmu.2023.1135489 PMC10154785 37153589

[19] Havel JJ Chowell D Chan TA. The evolving landscape of biomarkers for checkpoint inhibitor immunotherapy. Nat Rev Cancer. (2019) 19(3):133–50. doi: 10.1038/s41568-019-0116-x PMC6705396 30755690

[20] Zhou WT Jin WL. B7-H3/CD276: an emerging cancer immunotherapy. Front Immunol (2021) 12:701006. doi: 10.3389/fimmu.2021.701006 34349762 PMC8326801

[21] Scott EN Gocher AM Workman CJ Vignali DAA. Regulatory T cells: barriers of immune infiltration into the tumor microenvironment. Front Immunol (2021) 12:702726. doi: 10.3389/fimmu.2021.702726 34177968 PMC8222776

[22] Bartkowiak T Curran MA. 4-1BB agonists: multi-potent potentiators of tumor immunity. Front Oncol (2015) 5:117. doi: 10.3389/fonc.2015.00117 26106583 PMC4459101

[23] Curran MA Kim M Montalvo W Al-Shamkhani A Allison JP. Combination CTLA-4 blockade and 4-1BB activation enhances tumor rejection by increasing T-cell infiltration, proliferation, and cytokine production. PloS One (2011) 6(4):e19499. doi: 10.1371/journal.pone.0019499 21559358 PMC3085474

[24] Verbrugge I Hagekyriakou J Sharp LL Galli M West A McLaughlin NM. Radiotherapy increases the permissiveness of established mammary tumors to rejection by immunomodulatory antibodies. Cancer Res (2012) 72(13):3163–74. doi: 10.1158/0008-5472.CAN-12-0210 22570253

[25] Li H Borrego F Nagata S Tolnay M. Fc receptor-like 5 expression distinguishes two distinct subsets of human circulating tissue-like memory B cells. J Immunol (2016) 196(10):4064–74. doi: 10.4049/jimmunol.1501027 27076679

[26] Rincon-Arevalo H Wiedemann A Stefanski AL Lettau M Szelinski F Fuchs S. Deep phenotyping of CD11c(+) B cells in systemic autoimmunity and controls. Front Immunol (2021) 12:635615. doi: 10.3389/fimmu.2021.635615 33777025 PMC7994903

